# Evaluation of OspA Vaccination-Induced Serological Correlates of Protection against Lyme Borreliosis in a Mouse Model

**DOI:** 10.1371/journal.pone.0079022

**Published:** 2013-11-18

**Authors:** Michael G. Schwendinger, Maria O'Rourke, Andreas Traweger, Helga Savidis-Dacho, Andreas Pilz, Daniel Portsmouth, Ian Livey, P. Noel Barrett, Brian A. Crowe

**Affiliations:** Vaccine R&D, Baxter BioScience, Orth/Donau, Austria; University of Kentucky College of Medicine, United States of America

## Abstract

**Background:**

For clinical development of a novel multivalent OspA vaccine against Lyme borreliosis, serological assays are required which can be used to establish immune correlates of protection against infection with *Borrelia*.

**Methods:**

Four assays (an OspA IgG ELISA, a competitive inhibition (CI) ELISA, a *Borrelia* surface-binding (SB) assay and a *Borrelia* killing assay) were used to evaluate the correlation between immune responses induced by rOspA 1/2 (a chimeric immunogen containing protective epitopes from OspA serotypes 1 and 2), and protective immunity against infection by *B. burgdorferi* s.s. (OspA-1) and *B. afzelii* (OspA-2). Mice were immunized with OspA 1/2 doses ranging from 0.3 ng to 100 ng, to induce a range of OspA antibody titers, and exposed to needle challenge with *B. burgdorferi* s.s. or tick challenge with *B. afzelii*. Receiver operator characteristics (ROC) curves were constructed for each assay, and the area under the curve (AUC), sensitivity, specificity and Youden Index were calculated. Potential cutoff antibody titers which could be used as correlates of vaccine-induced protection were derived from the maximum Youden Index.

**Results:**

Immunization with OspA-1/2 provided dose-dependent protection against infection with *B. burgdorferi* s.s. and *B. afzelii*. Antibody responses detected by all four assays were highly significantly correlated with protection from infection by either *B. burgdorferi* s.s. (p<0.0001 to 0.0062) or *B. afzelii* (p<0.0001). ROC analyses of the diagnostic effectiveness of each assay showed the AUC to range between 0.95 and 0.79, demonstrating that all assays distinguish well between infected and non-infected animals. Based on sensitivity, specificity and AUC, the OspA IgG ELISA and SB assays best discriminated between infected and non-infected animals.

**Conclusions:**

All four assays differentiate well between *Borrelia*-infected and non-infected animals. The relatively simple, high throughput IgG ELISA would be suitable to establish immune correlates of protection for the novel OspA vaccine in clinical trials.

## Introduction

Lyme borreliosis (LB) is a multi-system inflammatory disease involving the skin, joints, heart, and nervous system [1], [2]. Approximately 30,000 LB cases are reported annually in the US [3], where it accounts for more than 95% of all reported cases of vector-borne illness [2], and approximately 85,000 LB cases occur annually in Europe [4]. LB can usually be treated with antibiotics [1], [2], but patients may remain unaware that they are infected until the onset of severe disease symptoms [1]. In the US, about 60% of non-treated infections cause Lyme arthritis, and about 10% of patients develop antibiotic-refractory arthritis [1]. LB is caused by bacteria of the *Borrelia burgdorferi* sensu lato (s.l.) species complex. Four species, *B. burgdorferi* sensu stricto (s.s.), *B. afzelii, B. garinii and B. bavariensis* cause the majority of human disease in Europe [5], whereas only a single species, *B. burgdorferi* s.s., causes LB in the US [1], [2].

Monovalent recombinant vaccines based on bacterial outer-surface protein A (OspA) serotype–1 derived from *B. burgdorferi* s.s were demonstrated to be safe and effective in clinical trials in the United States [6], [7], one of which (LYMErix) was licensed for human use in 1998. However, a non-substantiated hypothesis that LYMErix triggered arthritis in some vaccine recipients [8], [9] was one of a number of factors which contributed to the limited acceptance and subsequent discontinuation of the vaccine [10], [11]. Moreover, the monovalent OspA-1 vaccine did not have the potential to protect against LB outside of the US. Whereas *B. burgdorferi* s.s. only expresses OspA-1, *Borrelia* causing disease in Europe and Asia express a number of different OspA antigens ([1], [2]). Since immunity induced by OspA is largely type-specific [12]–[14], a multivalent OspA vaccine is required to prevent LB in these geographies.

To address this unmet need, we have initiated clinical studies of a novel multivalent OspA vaccine containing protective epitopes from OspA serotypes 1–6, to prevent LB in the US, Europe, and, possibly, globally [15], [16]. In pre-clinical proof-of principle studies, the bivalent OspA-1/2 component of the novel multivalent vaccine protected 100% of immunized mice from needle or tick challenge with *B. burgdorferi* s.s.(OspA-1) and *B. afzelii* (OspA-2), respectively [17].

For clinical development of the multivalent OspA vaccine, as well as for quality control, potency and stability testing, assays are required which enable the prediction of vaccine efficacy via the measurement of vaccine-induced immune responses. The primary mode of action of OspA antibody-mediated immunity is unusual in that it occurs in the mid-gut of the feeding tick rather than in the vaccine recipient. OspA antibodies are thought to prevent *Borrelia* infection by a number of mechanisms including direct or complement-mediated killing, growth inhibition, aggregation, or interference with one of the many specific functions attributed to OspA, such as plasminogen-binding promoting bacterial dissemination [18], [19], binding of TROSPA in the tick gut [20], [21], and protection from acquired host immunity [22], all of which require the recognition and binding of OspA.

A number of assays have previously been described which were designed to quantify protective OspA antibodies. OspA IgG antibodies induced by immunization with monovalent OspA-1 vaccines were reported to be predictive of protection in clinical trials [23]–[26], and an absolute correlate of protection against *B. burgdorferi* s.s. was established based on OspA-1 IgG ELISA titers [27]. The ability of OspA antibodies to inhibit *Borrelia* growth was also reported to be predictive of protection in humans [28] and animals [14], [29], [30]. In addition, monoclonal antibodies directed against characterized individual OspA-1 epitopes were shown to correlate with protection in mice and humans [7], [31]–[33]. The binding of antibodies to the surface of living *Borrelia* has also been described for the detection of immune responses to infected animals [34], [35], but this method has not previously been used to evaluate vaccine-induced antibody responses. Moreover, most of these earlier reports were restricted to the detection of OspA-1 antibodies, and it is not clear if these previously published data can be translated to other OspA serotypes.

In the present study, we evaluated the potential of four assays – an OspA IgG ELISA, a competitive inhibition (CI) ELISA, a surface-binding (SB) assay, and a killing assay – to predict vaccine-induced protection against infection with *B. burgdorferi* s.s. (OspA serotype-1) and *B. afzelii* (OspA serotype-2) in mice immunized with a bivalent OspA-1/2 immunogen.

## Materials and Methods

### Ethics Statement

All animal experiments were reviewed by the Baxter Bioscience Institutional Animal Care and Use Committee (IACUC Vienna/Orth) and approved by internal animal welfare officers. Animal experiments were conducted in accordance with Austrian laws on animal experimentation and approved by Austrian regulatory authorities (permit number LF1 TVG-38/009-2011). Experiments were conducted according to guidelines set out by the Association for Assessment and Accreditation of Laboratory Animal Care International (AAALAC). Animals were housed according to EU guidelines, in housing facilities accredited by the AAALAC. For blood sampling and challenge, all animals were anaesthetized with isoflurane using UNO – Univentor Anaesthesia Unit according to manufacturer's protocol. All efforts were made to minimize suffering.

### Borrelia and ticks

*B. burgdorferi* s.s. strains ZS7 and B31 (expressing OspA-1), and *B. afzelii* strain Arcon (OspA-2) were incubated at 33°C with 5% CO_2_ in a modified Barbour Stoenner Kelly medium (BSK-B) [36]. *B. afzelii*-infected nymphal ticks were either collected in the field near Budweis in the Czech Republic, an area where LB is endemic, or purchased from IS Insect Services GmbH (Berlin). The rate of B. *afzelii* infection in infected ticks, as determined by qPCR [36], was 27% in feral ticks and >50% in laboratory-reared ticks.

### Immunization and challenge

Groups of 8–15 female C3H/HeOuJ mice (Charles River) were immunized subcutaneously with OspA-1/2 antigen [17], adjuvanted with 0.2% Al(OH)_3_, at doses ranging from 0.3 ng to 100 ng total antigen, or buffer control. Mice received one or two immunizations, 28 days apart. Animals receiving a single immunization were challenged 21 days post-immunization; animals receiving two immunizations were challenged 14 days post-immunization. For needle challenge with *B. burgdorferi* s.s. ZS7, mice were injected intradermally with 10^4^ bacteria. For tick challenge with *B. afzelii*, 8 feral or 4 laboratory-reared nymphal ticks were applied to each mouse 14 days after immunization and allowed to feed for 5 to 6 days before removal. Blood was obtained from each mouse one day before challenge. Mice were sacrificed four weeks post-challenge. Infectious status was determined by C6-peptide ELISA, western blotting, culture, and real time PCR, as described previously [17]. Mice, which were positive by any one of 3 methods, namely, by serology (C6 ELISA or Western Blotting) or by PCR or by culture, were considered infected.

### IgG ELISA

Recombinant OspA antigens derived from *B. burgdorferi* s.s. strain B31 (OspA-1) and *B. afzelii* strain ACA1 (OspA-2) were expressed as GST-OspA fusion proteins in *E.coli*, purified by affinity chromatography and used to coat ELISA plates at 1 µg/ml. Dilutions of pre-challenge sera or a serial dilution of OspA monoclonal antibody (mAb) with defined IgG concentration were added to the plates. After incubation with a peroxidase-conjugated goat anti-mouse detection antibody (Jackson) and development with TMB substrate (Becton Dickinson), anti-OspA IgG values (µg/ml) were calculated.

### Competitive inhibition ELISA

Monoclonal antibodies F237/AE2 and F237/BV3 were generated by immunizing C3H/HeJ mice with OspA-1/2 and selected based on binding specificity to OspA proteins and live *Borrelia*, as well as protective efficacy against *Borrelia* infection in mouse passive protection experiments. F237/AE2 binds specifically to OspA-1 and *B. burgdorferi* s.s. bacteria and provides complete protection against needle challenge with *B. burgdorferi* s.s. F273/BV3 binds specifically to OspA-2 and *B. afzelii* bacteria, and provides complete protection against tick challenge with *B. afzelii*. Plates were coated with 1 µg/ml GST-OspA-1 or GST-OspA-2. After blocking with TBS/Tween/BSA, test sera were applied. Serial dilutions of non-labeled mAb served as standard. After 60 mins incubation, peroxidase-labeled mAbs were added to all wells for a further 45–60 mins. Plates were developed with TMB substrate, and relative mAb equivalent concentrations were calculated as ELISA units/ml (ELU).

### Surface-Binding (SB) Assay

Approximately 3×10^6^ *B. burgdorferi* s.s. strain B31 (OspA-1) or *B. afzelii* strain Arcon (OspA-2) bacteria were incubated with immune (pre-challenge) mouse sera at a dilution of 1∶100, or serial dilutions of a standard mouse serum pool with defined SB titer (inverse of the dilution exhibiting >3× fluorescence intensity over background). After washing, bound antibodies were labeled by incubating for 10 mins with 5 µg/ml r-Phycoerythrin-conjugated anti-mouse Ig polyclonal antibody (Southern Biotech). 2.5 µg/ml DNA stain LDS-751 (Molecular Probes) was added, and bacteria were analyzed by flow cytometry (FACSCalibur, BD BioScience). FlowJo software (Tristar) was used to gate labeled *Borrelia* according to scatter and fluorescence intensity, and relative SB titers were calculated.

### Killing Assay

Serial dilutions (1∶3) of heat-inactivated serum samples were incubated in the presence of guinea pig- or baby rabbit-derived complement with *B. burgdorferi* s.s. strain B31 or *B. afzelii* strain Arcon. After 3–5 days, the BacTiter-Glo™ Microbial Cell Viability assay (Promega) was utilized to determine the relative concentrations of viable *Borrelia*. K50 titers were determined according to the reciprocal of the highest serum dilution which resulted in >50% reduction in *Borrelia* viability.

### Statistical Analyses

Comparision of antibody titers in infected and protected animals using the Mann-Whitney U test, and calculation of receiver operator characteristics (ROC) and area under the curve (AUC) were done using Graphpad Prism Vers. 5.0. Thresholds for antibody levels optimally discriminating between protected and infected mice were chosen such that the respective Youden Index was maximal Sensitivity (fraction of infected animals with antibody levels < the cutoff) and Specificity (fraction of non-infected animals with antibody levels ≥ the cutoff) were calculated using Microsoft Excel 2007.

## Results

### Dose-dependent protection against infection with *B. burgdorferi* s.s and *B. afzelii*

The relationship between OspA vaccination-induced antibody responses, induced by different immunization doses and schedules, and protective immunity in mouse challenge models are shown in Table 1 for the IgG ELISA, CI-ELISA, SB assay and killing assay. A wide range of antibody responses was obtained for assays measuring OspA-1 antibodies (upper panel) as well as for the assays measuring OspA-2 antibodies (lower panel).

**Table 1:** GMT antibody titers and protection of vaccinated mice against challenge with *B. burgdorferi* s.s. and *B. afzelii*.

	Antibody GMT				
	IgG ELISA (µg/mL)	CI-ELISA (ELU)	SB assay (dil^−1^)	Killing assay (dil^−1^)	Infected mice (n/N)
Needle challenge with *B. burgdorferi* s.s. (OspA-1)					
**OspA 1/2 dose (ng)**					
1×100	19	21	1938	394	0/15
1×30	8.5	9	698	128	1/15
1×10	1.1	4.7	73	21	4/14
2×30	64	53	1841	805	0/10
2×10	20	26	240	57	0/10
2×3	4.3	15	96	38	2/10
2×1	1.5	3.4	37	24	2/10
none	0.2	0.2	14	11	19/20
Tick challenge with *B. afzelii* (OspA-2)					
**OspA 1/2 dose (ng)**					
2×100	150	130	320	2839	1/26
2×30	53	122	164	2200	3/26
2×3	1.0	66	15	24	8/10
2×0.3	0.2	48	7	14	10/10
none	0.2	28	7	11	16/18

Immunization with OspA-1/2 provided dose-dependent protection against *B. burgdorferi* s.s. (Table 1, upper panel) and *B. afzelii* (Table 1, lower panel). Mice which received two immunizations with a 30 ng or 10 ng dose or one immunization with a 100 ng dose were completely (100%) protected against infection from a needle challenge with *B. burgdorferi* s.s. In contrast, 19 of 20 animals receiving buffer alone were infected. Higher antigen doses and two immunizations were needed to provide substantial protection against tick challenge with *B. afzelii* (Table 1, lower panel). Protection was almost complete in animals receiving the 100 ng and 30 ng doses (96% and 88%, respectively), whereas infection was seen at lower doses or in animals receiving buffer only.

### Correlation of antibody titers with protection against *B. burgdorferi* s.s

**Figure 1:**
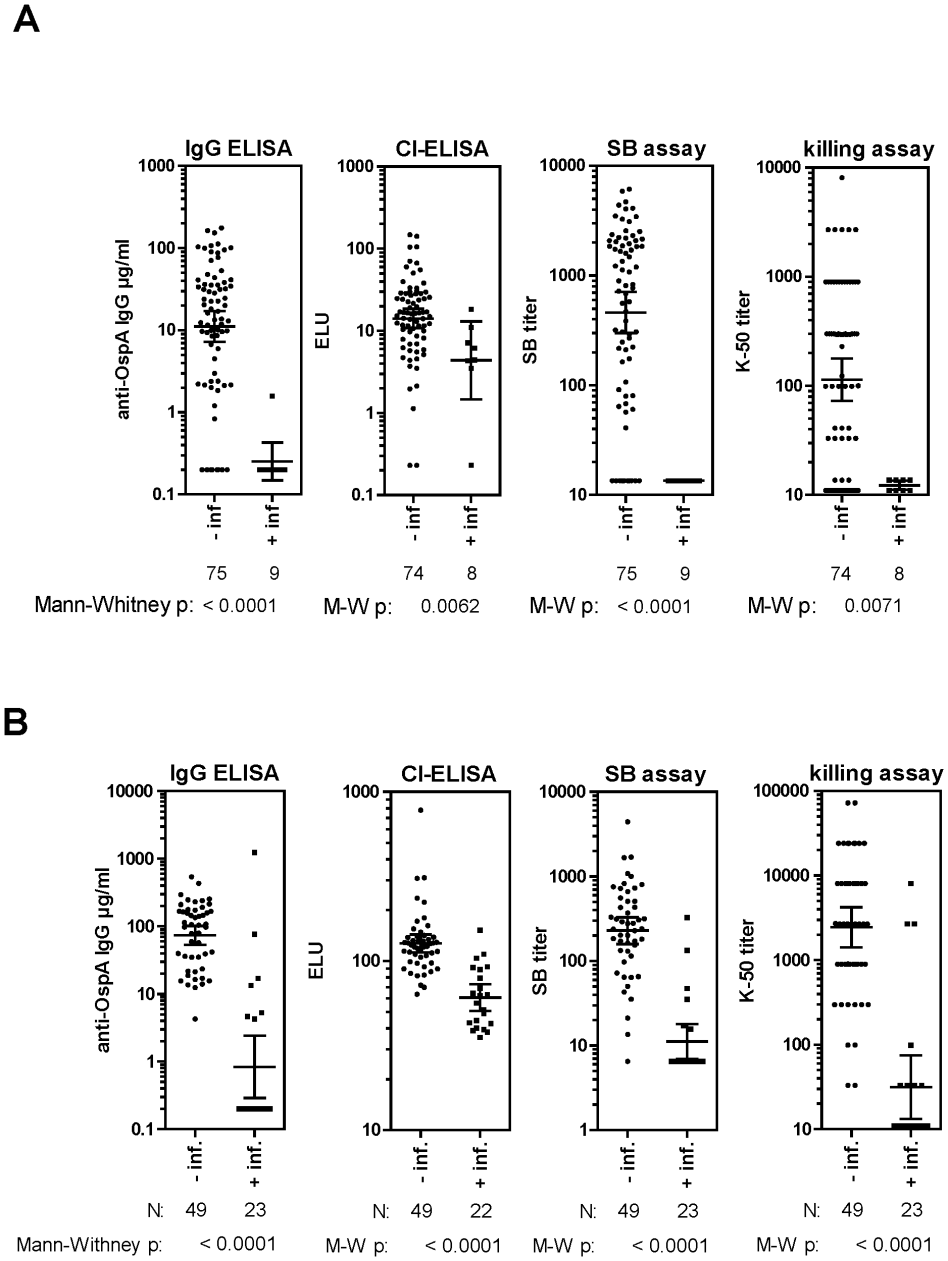
Correlation of immunological assays with rOspA-1/2 vaccine-induced protective efficacy. Serum antibody titers quantified by ELISA, CI-ELISA, SB assay and killing assay are shown for OspA-1/2-immunized mice which were either protected from or infected by (**A**) needle challenge with *B. burgdorferi s.s.* strain ZS7 or (**B**) tick challenge with *B. afzelii*. Shown are the geometric mean titers of each group as well as individual serum titers. P values represent the strength of the correlation of assay titers with protective efficacy as calculated by the Mann-Whitney U test. Two serum samples were unavailable for evaluation in the *B. burgdorferi* s.s CI and killing assays; one serum sample was unavailable for evaluation in the *B. afzelii* CI assay.

The correlation between OspA-1/2 vaccination-induced protection against *B. burgdorferi* s.s. and antibody titers determined by the OspA-1 IgG ELISA, CI-ELISA, SB and killing assays were evaluated. Scatter plots depicting the antibody responses of individual mice grouped according to infection status are shown in Figure 1A. Of 84 mice immunized with varying OspA 1/2 antigen doses, 75 (89%) were protected against needle challenge with *B. burgdorferi* s.s., and 9 (11%) were infected. GMT antibody titers determined in protected and infected mice by each of the four assays are shown in Table 2, upper panel. GMTs in non-infected mice were 3-fold (CI-ELISA) to 55-fold (IgG ELISA) higher than GMTs in infected mice. Statistical comparison of the two groups (Mann-Whitney U test) showed that these differences were highly significant (p<0.0001 to 0.0062). The GMT differences between infected and non-infected mice were most significant for the IgG ELISA and SB assay (p<0.0001).

**Table 2:** Antibody GMTs in infected and non-infected mice; sensitivity, specificity, AUC and maximum Youden Index for each assay; and cutoff titers derived from the Youden Index which could be used as correlates of protection.

	IgG ELISA (µg/mL)	CI-ELISA (ELU)	SB assay (dil^−1^)	Killing assay (dil^−1^)
Needle challenge with *B. burgdorferi* s.s. (OspA-1)				
GMT non-infected	11	14	461	114
GMT infected	0.2	4	14	12
P value protected vs infected	<0.0001	0.0062	<0.0001	0.0071
AUC	0.94	0.80	0.90	0.79
Sensitivity	1.00	0.88	1.00	1.00
Specificity	0.87	0.62	0.87	0.69
Max. Youden Index	0.87	0.50	0.87	0.69
Cutoff titer	1.7	12	30	20
Tick challenge with *B. afzelii* (OspA-2)				
GMT non-infected	73	127	228	2445
GMT infected	0.8	61	11	31
P value protected vs infected	<0.0001	<0.0001	<0.0001	<0.0001
AUC	0.93	0.91	0.95	0.89
Sensitivity	0.83	0.73	0.91	0.87
Specificity	0.98	0.94	0.90	0.92
Max. Youden Index	0.81	0.67	0.81	0.79
Cutoff titer	10	80	50	100

**Figure 2:**
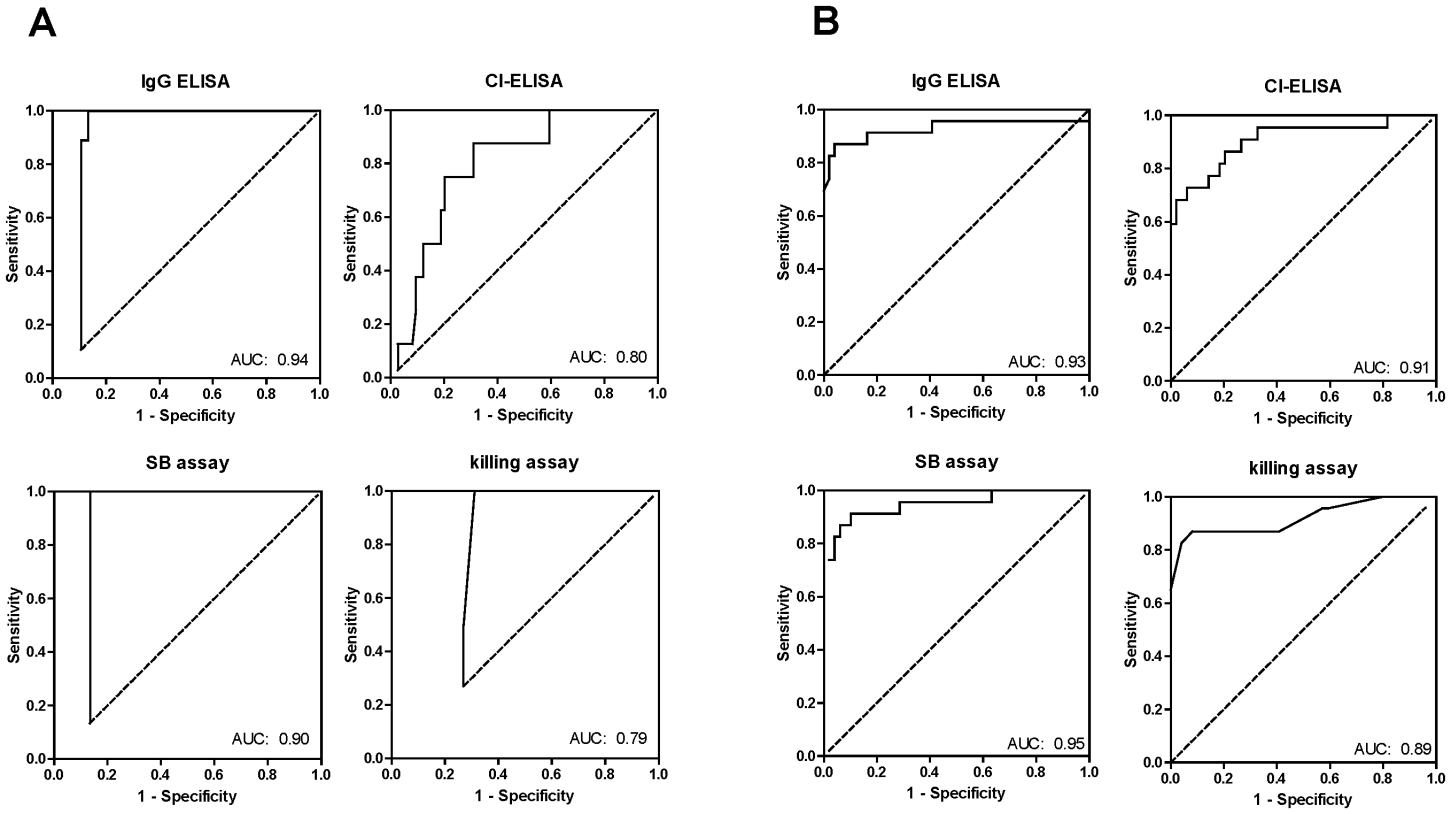
ROC curve analysis of four immunological assays. Assays were evaluated for their ability to discriminate non-infected from infected mice in challenge experiments using (**A**) *B. burgdorferi* s.s. and (**B**) *B. afzelii*. Area under the curve (AUC) values are included.

ROC curves were constructed for each assay (Figure 2A), and the AUC was calculated, which indicates the diagnostic effectiveness of the assay, i.e. the ability to distinguish between infected and non-infected animals. ROC analyses showed the AUC to range between 0.94 (IgG ELISA) to 0.79 (killing assay) (Table 2, upper panel), demonstrating that all assays distinguish well between infected and non-infected animals, albeit the AUC was higher for the OspA-1 IgG ELISA and SB assay compared to the CI and killing assays.

The sensitivity, specificity and Youden Index (sensitivity + specificity −1) were calculated for each assay (Table 2, upper panel). The sensitivity (1.0) and specificity (0.87) of the IgG ELISA and SB assays were both high, whereas the specificity of the CI-ELISA and the killing assay were somewhat lower, as was the sensitivity of the CI-ELISA. Derived from the maximal Youden Index (where the sum of specificity and sensitivity is maximized) individual cutoff titers were determined which could be used as correlates of vaccine-induced protection against needle challenge with *B. burgdorferi* s.s. (Table 2, upper panel). These occurred at titers of 1.7, 12, 30 and 20 for the OspA-1 IgG ELISA, CI-ELISA, SB assay and killing assay, respectively.

### Correlation of antibody titers with protection against *B. afzelii*

Scatter plots depicting the responses of the individual mice grouped according to *B. afzelii* infection status are shown in Figure 1B. Of 72 mice immunized with varying OspA 1/2 antigen doses, 49 (68%) were protected against tick challenge with *B. afzelii* and 23 (32%) were infected. The differences in antibody responses determined for the protected compared to the infected mice were statistically highly significant for all four assays (p<0.0001). GMTs measured in protected and infected mice for each of the four assays are shown in Table 2 (lower panel). The fold increases in GMTs of protected versus infected mice varied considerably for each of the assays (91-fold for the IgG ELISA; 79-fold for the killing assay; 21-fold for the SB assay; and 2-fold for the CI-ELISA).

ROC analyses performed on each assay (Figure 2B) demonstrated the AUC to be high for all four assays (0.89 to 0.95), indicating that each assay was effective at distinguishing between *B. afzelii*-infected and non-infected mice (Table 2). Specificity was high for all assays (>0.90); however the sensitivities of the OspA-2 IgG ELISA, SB assay and killing assay were slightly higher than those for the CI-ELISA. Cutoff titers, derived from the maximum Youden Index, which could be used as correlates of protection against tick challenge with *B. afzelii*, occurred at titers of 10, 80, 50 and 100 for the OspA-2 IgG ELISA, CI-ELISA, SB assay and killing assay, respectively. One mouse infected with *B. afzelii* had titers substantially above the calculated thresholds of protection. Antibody titers in this mouse, determined by all four assays, were considerably higher than the calculated threshold values, i.e. none of the assays was able to predict this breakthrough infection.

## Discussion

We investigated the relationship between OspA vaccination-induced antibody responses and protective immunity in mouse challenge models using four different assays: an IgG ELISA, a CI-ELISA, a SB assay and a killing assay. All four assays were highly predictive of protection against needle challenge with *B. burgdorferi* s.s. (OspA-1) and tick challenge with *B. afzelii* (OspA-2); however, the IgG ELISA and SB assay were more specific and more sensitive than the killing assay or CI-ELISA, and were better at discriminating protected from infected mice. This conclusion is supported by the slightly higher AUC values observed for the IgG ELISA and SB assays in the ROC analysis. The higher sensitivity and specificity of the IgG ELISA and SB assays might be explained by the fact that surface-binding responses include a wider range of antibodies and epitopes than those involved in bactericidal or specific protective epitope responses. The finding that OspA-1 and OspA-2 IgG antibody ELISA titers were highly predictive of protection, despite the fact that a proportion of detected OspA antibodies are presumably non-functional and have no relevance to protection, indicates that the proportion of protective OspA antibodies of the total OspA antibodies induced by the OspA-1/2 vaccinogen is large and/or relatively constant.

In general, our data are in agreement with studies which reported that IgG ELISA [23]–[26], CI-ELISA [31]–[33] and killing assays [28], [31], [32] are able to distinguish between infected and non-infected animals and humans. To our knowledge, however, a surface-binding assay has not previously been used to specifically and sensitively predict OspA-mediated vaccine-induced protection against *Borrelia* infection. In contrast to our data, some previously published studies of antibody responses induced by monovalent OspA-1 antigen or whole *B. burgdorferi* s.s have reported that a killing assay was more sensitive and/or specific than an IgG ELISA, and might be a better predictor of protection against infection [37], [38]. A possible explanation for the different conclusions of these previous studies could be that the rOspA-1/2 antigen used in the present study, in comparison to the native OspA-1 antigen used in previous studies, induces a higher and more constant proportion of specific antibodies capable of killing *Borrelia*.

In the present study, the relative levels of antibodies required for protection against tick challenge with *B. afzelii* were higher than required to protect against needle challenge with *B. burgdorferi* s.s. However, it is difficult to directly compare these data, due to the different challenge routes and potential differences in OspA expression level between the two strains used in the challenge studies.

Despite very high antibody titers, one immunized mouse was infected with *B. afzelii* following feral tick challenge. It is unclear why this animal was not protected; one explanation might be that *Borrelia* were already present in the salivary gland of the unfed tick, or the tick was systemically infected, such that they were no longer susceptible to OspA antibodies [39]–[41]. Antibody titers determined by all four assays were considerably higher than the calculated cutoff values, suggesting that none of the evaluated assays were able to predict this infection. However, the occurrence of only a single breakthrough infection among a total of 156 challenged mice indicates that all four assays provide excellent discrimination between protected and infected animals, and could be used to define absolute correlates of vaccine-induced protection against infection by both *B. burgdorferi* s.s. and *B. afzelii*. In this respect, our data suggest that the IgG ELISA and the SB assays are slightly more sensitive and more specific compared to the CI-ELISA or the killing assay.

Further studies are in progress to determine if the conclusions of the present study, for OspA-1 and OspA-2 antibodies, also translate to antibodies directed against the other OspA components of the novel multivalent OspA vaccine, i.e. OspA serotypes 3–6. Phase I/II studies with the multivalent vaccine have been initiated in humans to determine the optimal dose, formulation, and booster interval in *Borrelia* seronegative as well as *Borrelia* seropositive individuals [15], [16]. Serological assays established in the present study will ultimately be used to determine clinical correlates of protection as part of a phase III efficacy study.
